# Polycystic liver disease: an overview of pathogenesis, clinical manifestations and management

**DOI:** 10.1186/1750-1172-9-69

**Published:** 2014-05-01

**Authors:** Wybrich R Cnossen, Joost PH Drenth

**Affiliations:** 1 Department of Gastroenterology and Hepatology, Institute for Genetic and Metabolic Disease, Radboud university medical center, Geert Grooteplein-Zuid 10, P.O. Box 9101, 6525 GA Nijmegen, The Netherlands

**Keywords:** Cystogenesis, Polycystic liver disease (PLD), Hepatomegaly, Biliary tract disease, Ductal plate malformation (DPM), Von Meyenburg complex (VMC), Autosomal dominant polycystic liver disease (PCLD), Autosomal dominant polycystic kidney disease (ADPKD)

## Abstract

Polycystic liver disease (PLD) is the result of embryonic ductal plate malformation of the intrahepatic biliary tree. The phenotype consists of numerous cysts spread throughout the liver parenchyma. Cystic bile duct malformations originating from the peripheral biliary tree are called Von Meyenburg complexes (VMC). In these patients embryonic remnants develop into small hepatic cysts and usually remain silent during life. Symptomatic PLD occurs mainly in the context of isolated polycystic liver disease (PCLD) and autosomal dominant polycystic kidney disease (ADPKD). In advanced stages, PCLD and ADPKD patients have massively enlarged livers which cause a spectrum of clinical features and complications. Major complaints include abdominal pain, abdominal distension and atypical symptoms because of voluminous cysts resulting in compression of adjacent tissue or failure of the affected organ. Renal failure due to polycystic kidneys and non-renal extra-hepatic features are common in ADPKD in contrast to VMC and PCLD. In general, liver function remains prolonged preserved in PLD. Ultrasonography is the first instrument to assess liver phenotype. Indeed, PCLD and ADPKD diagnostic criteria rely on detection of hepatorenal cystogenesis, and secondly a positive family history compatible with an autosomal dominant inheritance pattern. Ambiguous imaging or screening may be assisted by genetic counseling and molecular diagnostics. Screening mutations of the genes causing PCLD (*PRKCSH* and *SEC63*) or ADPKD (*PKD1* and *PKD2*) confirm the clinical diagnosis. Genetic studies showed that accumulation of somatic hits in cyst epithelium determine the rate-limiting step for cyst formation. Management of adult PLD is based on liver phenotype, severity of clinical features and quality of life. Conservative treatment is recommended for the majority of PLD patients. The primary aim is to halt cyst growth to allow abdominal decompression and ameliorate symptoms. Invasive procedures are required in a selective patient group with advanced PCLD, ADPKD or liver failure. Pharmacological therapy by somatostatin analogues lead to beneficial outcome of PLD in terms of symptom relief and liver volume reduction.

## Review

### Disease name

Polycystic liver disease (PLD) is a collection of rare human disorders that result from structural changes in the biliary tree development [1,2]. Genetic mechanisms and/or signaling defects are the root cause of ductal structures to become separated from the biliary tree finally resulting in cyst formation [2,3]. Typically, these disconnected biliary structures are present in a very early disease stage, but remain asymptomatic until cyst growth initiates in adulthood [4].

Three PLD entities are recognized in adults. Von Meyenburg complexes (VMC; biliary hamartoma; hepatic cystic hamartoma) with characteristic small, non-hereditary nodular cystic lesions [ORPHA386] [4,5]. Isolated polycystic liver disease (PCLD; autosomal dominant PLD) [OMIM#174050; ORPHA2924] with presence of innumerable hepatic cysts and autosomal dominant polycystic kidney disease (ADPKD) [OMIM#173900; OMIM#613095; OMIM#600666; ORPHA730] with cysts in both kidneys and in many cases hepatic cysts.

This paper reviews the pathological and clinical features of these 3 adult cystic disorders that share presence of numerous hepatic cysts with an intact biliary tree architecture.

### Definition

PLD is a rare inherited Mendelian disorder that is characterized by development of multiple hepatic cysts. The classification of PLD follows the histological changes that are due to ductal plate malformation (DPM) during fetal development [6,7]. Definitions of cystic malformations are either based on the location of the affected (cilium-related) protein or follows radiological findings [8,9].

### Ductal plate malformation

The biliary tree emerges from the endodermal hepatic diverticulum [1]. Development of the biliary system starts from the 8^th^ week of gestation by formation of single layered hepatoblasts surrounding the portal vein (ductal plate). Duplication of ductal plate cells forms a double layer that finally dilate to a tubular structure, the primitive bile duct. Hepatoblast differentiation to a biliary phenotype and tubulogenesis is stimulated by the Notch, TGF-β and canonical Wnt signaling pathways [2]. Cell differentiation from hepatoblasts to cholangiocytes, tubule elongation and bile duct remodeling are completed by 30 weeks of gestation. Intrahepatic and extrahepatic bile duct systems are then merged and share the hepatic hilum. During the first year of life intrahepatic biliary epithelium maturates further [1,2,7]. PLD develops as a result of ductal plate malformation. The stage that is affected by faulty remodeling determines the phenotype. For example, VMC is thought to result from embryonic ductal involution at a late stage [7,10].

Bile duct formation requires a network of epithelial-mesenchymal interactions, and presence of growth and transcription factors to control appropriate cell migration, adhesion and cholangiocyte differentiation. Aberrant expression profiles and signaling result in deficient remodeling, and subsequently abnormal dilated or disconnected ductal plate cells developing into biliary cystic structures [3,11]. Recently, a new classification for DPM has been proposed on the basis of *Hnf-1*β, *Hnf-6* and *cystin-1* gene deficient mouse models. This classification distinguishes 3 DPMs: 1) abnormal hepatoblast differentiation, 2) failure of bile duct maturation, 3) perturbation of ductal expansion [6].

### Ciliopathy and cholangiopathy

Ciliopathies represent an emerging class of human disorders that are caused by defects in distinct genes affecting ciliary structures or function. They may be inherited as simple recessive traits, but also in a dominant fashion. Phenotypic expressivity is under the control of numerous genetic modifiers [8]. Ciliopathies usually result in shared clinical features, such as intellectual disability, retinal defects and polydactyly, but the most well-known phenotype is that of cystic kidneys [12]. The proteins affected in ADPKD are located at the cilium which has led to the classification of ADPKD as a ciliopathy [13]. By contrast, the proteins associated with PCLD are not located to the cilium. Hepatic cysts are lined by cholangiocytes and therefore the term cholangiopathy is used for PCLD [3].

### Radiology

**Figure 1:**
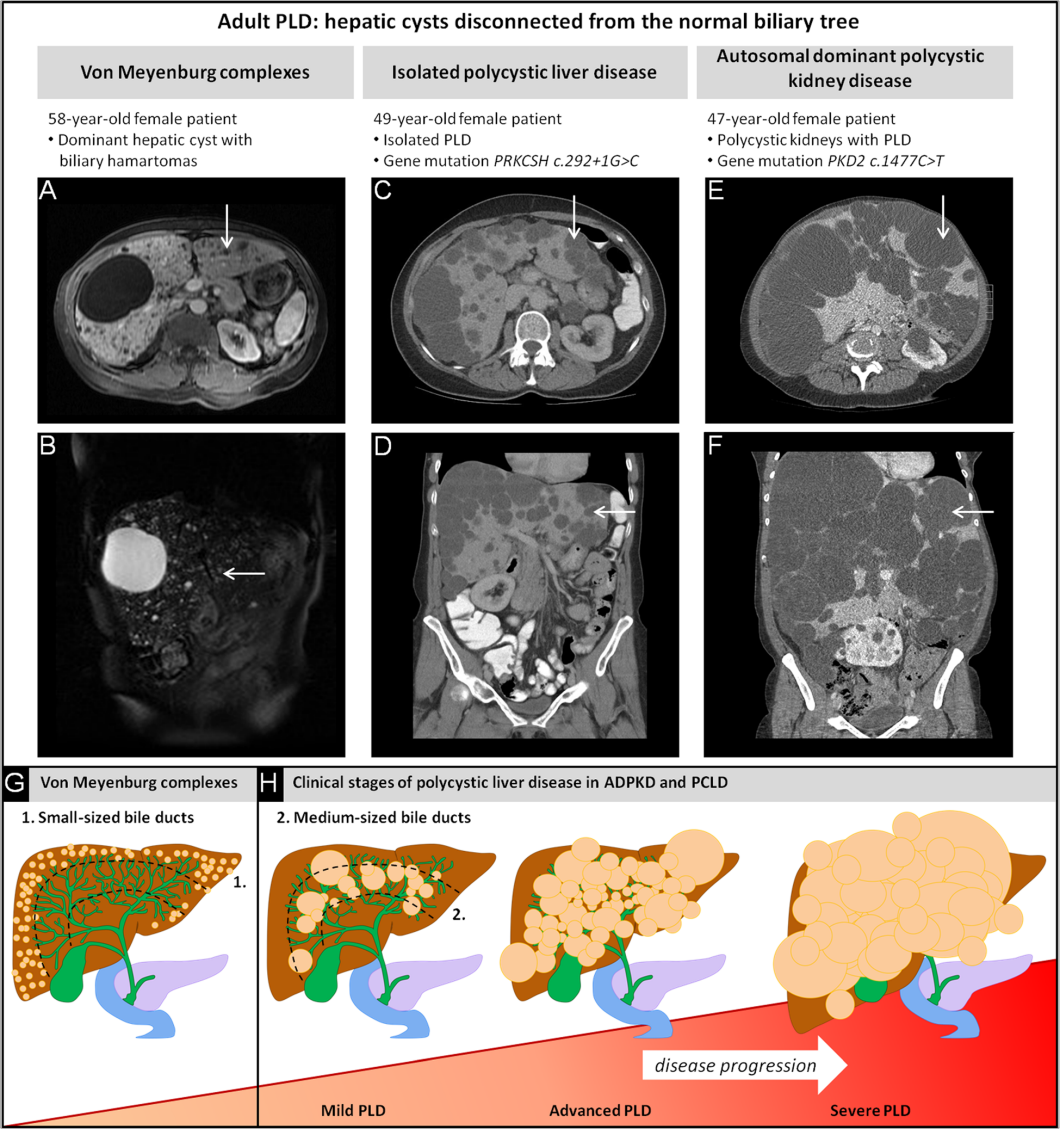
**Abdominal MRI and CT in patients with PLD. (A)** Axial T1-weighted and **(B)** coronal T2-weighted MRI present 1 large cyst and numerous cystic nodules scattered at peripheral bile ducts. **(C-D)** CT-scanning in a PCLD patient presents multiple cysts originating from medium-sized bile ducts. **(E-F)** Co-occurrence of polycystic kidneys exists in ADPKD. Both *PRKCSH* and *PKD2* gene mutations were predicted to be pathogenic (GRCh37-hg19; HGMD). Hepatic cysts are indicated by white arrows. **(G)** Diffuse VMC present numerous small-sized hepatic cysts located at peripheral branches of the biliary tree (in green). **(H)** The PLD phenotypes are arbitrarily staged and indicate disease progression. The disease course is progressive in a subset of severely affected PLD patients.

Radiological imaging assists in classifying PLD. Detection of macroscopically hepatic and renal cysts is facilitated by ultrasonography, magnetic resonance imaging (MRI) or computed tomography (CT)-scanning without or with (creatinine-permitting) intravenous contrast material [9]. On ultrasound, cysts appear as homogeneous anechoic fluid-filled round spaces. MRI is superior over ultrasound and CT, and allows better detection of small cysts in young individuals [14]. This technique captures biliary tree pathology and differentiates parenchym from biliary tree (Figure 1).

### Epidemiology

PCLD has a prevalence of 1/100,000 to 1/1,000,000 or 1- 9/100,000 (1/158,000 in The Netherlands), while the prevalence of ADPKD ranges between 1/400 to 1/1,000 [13,15]. The incidence of VMC has been estimated up to 1/18-1/145 or 7-60/1,000 (0.69-5.6%) depending on the various autopsy studies [10,16].

### Clinical description

#### Von Meyenburg complexes

VMC, also termed microhamartomas, are benign cystic nodules scattered throughout the liver. They are usually interlobularly located and at peripheral bile ducts below the Glisson’s capsule [7]. VMC may occur isolated or in the context of PCLD and ADPKD [4,11,17]. VMC are frequently an incidental finding at radiological imaging, surgery or autopsy studies.

Histologically, they are characterized by small embryonic DPM remnants (<1.5-cm-diameter) or larger (small) hamartomas (>1.5 cm) delineated by regular cuboidal epithelium and embedded in fibrous stroma. Dilated structures initially communicate with the peripheral intrahepatic biliary tree, but separate with development [7,10].

VMC usually remain silent during life and require no management or follow-up examination [17]. Although mild liver test disturbances may be observed, significant hepatomegaly or liver disease is rare in VMC. Incidentally, clinical features of epigastric pain, fever, cholangitis and jaundice appear when communication of multicystic VMC with the biliary tree cause biliary obstruction [18]. Episodes of liver sepsis indicates antibiotic treatment and follow-up of liver function tests. Abdominal pain and discomfort resolves with time. The diagnosis of VMC can be confirmed by MRI. Extra-hepatic features are absent.

### PCLD and ADPKD

Hepatic cysts are the major clinical feature in PCLD and the most frequent extra-renal manifestation in ADPKD [14]. Cysts originate from medium-sized bile ducts. Histologically, they are delineated by cuboidal, flattened epithelial cells surrounded by fibrous stroma [7].

They may be confined to 1 or more segments or spread evenly throughout the liver. Presence of large and numerous cysts frequently lead to hepatomegaly. One study showed that the overall hepatic cyst prevalence in ADPKD patients (age range15-46 years) is 83%, with the highest prevalence of 94% in 35- to 46-year-old patients. This corresponds with an increased prevalence of multiple hepatic cysts in older PLD patients [14].

The variable number, size, location and distribution of cysts determines the spectrum of symptoms which is related to the extent of hepatomegaly [4]. Pain may ensue mainly because of tension on Glisson’s liver capsule. Abdominal discomfort, pyrosis, early satiety, weight loss and anorexia may arise in advanced PLD [15,19]. Patients who have large hepatic cysts that exert pressure on the stomach and displacement of other abdominal organs are at risk for malnutrition and nutritional deficiencies [20].

Females with ADPKD or PCLD usually have a more severe liver phenotype, especially for those with a history of multiple pregnancies and prolonged exogenous estrogen exposure [17,21,22]. Females have higher average liver volumes, are younger at presentation and more susceptible to progressive PLD, suggesting a hormonal component [14,15,19]. In general, patients with PCLD have more severe symptoms and liver-related complications compared to ADPKD [23]. Hepatic cysts are rarely present in young children, but in exceptional cases symptomatic PLD may develop in young ADPKD patients.

The risk to develop severe PLD is independent from the ADPKD genotype, but is related to the severity of renal disease [13,23]. Here, presence of a *PKD1* mutation puts the patient at risk for an earlier onset and outcome of renal disease compared to the *PKD2*[24,25].

### Hepatic complications in PCLD and ADKPD

Complications may occur due to massively hepatomegaly or result from invasive treatment [15,19]. It has been estimated that about half of the patients with advanced hepatic disease have had cyst hemorrhage, cyst rupture or a cyst infection [15,26]. These manifestations appear to be more frequent in ADPKD compared to PCLD [27]. Here, we will discuss the clinical and diagnostic signs for cyst hemorrhage, rupture, infection and other rare complications in PCLD and ADPKD.

#### Hemorrhage

Intrahepatic cyst bleeding typically presents as acute pain in the upper right-side of the abdomen. Symptoms develop acutely, progress in the first day(s), but resolve spontaneously. Sometimes, the nature of the abdominal pain can be colicky and accompanied by vomiting. Ultrasonography may be helpful to assess features suggestive for cyst bleeding such a higher attenuation value, aggregated fibrin deposits and possibly internal septa of hematomas. High signal intensity on MRI supports the diagnosis of an intrahepatic cyst bleeding [9,28].

#### Infection

Cyst infections are a serious complication because of its indolent course, demanding treatment and high risk of recurrence. Current diagnostic criteria rely on clinical, biological and radiological parameters including abdominal tenderness, fever (>38°C for >3 days), an increased C-reactive protein level and proven absence of spontaneous intra-cystic bleeding by CT-scanning. Hepatic cyst wall thickening and heterogeneous fluid (debris) are suggestive for infection. The diagnostic accuracy of MRI is unknown and CT-scanning has a low sensitivity and specificity to identify cyst infection [29]. ^18^ F-FDG positron emission tomography (PET)-imaging technique is preferable to detect the exact location of the infected lesion(s) [29].

PLD patients may have strongly elevated CA19-9 levels [30]. Extremely high CA19-9 levels were found in episodes with hepatic cyst infection and declining during recovery [31]. Detection of neutrophils and infectious agent(s) in the cyst fluid aspirate confirms the cyst infection and indicative for targeted antibiotic treatment [29].

#### Rupture

Cyst rupture is an exceptional rare complication and presents with acute onset of pain. Hemodynamic complications are rare, but have been reported in the literature [32]. Abdominal discomfort from prolonged ascites and increased cyst volume needs stringent follow-up. If intra-peritoneal (blood) fluid leakage persists, a surgical intervention is inevitable for hemostasis control [28].

#### Portal hypertension and ascites

In advanced stages there are 2 processes that may lead up to portal hypertension. First, there is reduction of hepatic vein outflow. Secondly, portal vein inflow may be compressed in advanced disease due to the volume effect of cysts.

Signs of hepatic vein outflow obstruction (HVOO) are abdominal pain, hepatomegaly and transudative ascites (90-96%) [33]. Hepatic vein thrombosis is commonly recognized as the cause of HVOO, and case series reported also the Budd-Chiari syndrome secondary to PLD [33,34]. The survival of hepatic vein thrombosis is low in severe cases [35]. Mechanical pressure symptoms of hepatomegaly may extend from hepatic veins to junctions with the vena cava inferior (IVC). Compression of IVC is characterized by increased renal outflow pressure that provokes development of ascites and edema in the lower extremities [36].

In addition, hepatic cysts may cause a compromised portal venous flow [37]. These abovementioned complications lead to development liver fibrosis. Secondary complications of portal hypertension are the result of severe liver fibrosis such as esophageal varices, splenomegaly and transudative ascites may develop, but advanced fibrosis is a rare event [26]. Typically these features are seen in the elderly. In addition, lymphatic leak and chronic renal disease may contribute to development of ascites in severe PLD patients [33,37].

#### Jaundice

Portal hypertension may be accompanied by other signs of hepatic failure such as jaundice [38]. Although jaundice is usually seen in advanced stages, it may occur at any stage. An uncommon cause of jaundice is obstruction of the intrahepatic or extrahepatic bile ducts by hepatic cysts [26]. Recurrent cholangitis is a rare complication of this condition [34].

#### End-stage liver disease

Progression to end-stage liver disease usually results in the context of extremely increased liver volumes. Liver failure is seen incidentally, usually in a very late stage of the disease [15,19]. Symptomatic patients with hepatomegaly (severe PLD) frequently do not meet the Model for End-Stage Liver Disease (MELD). Therefore, MELD exception criteria including assessment of malnutrition and quality of life are used [20]. Liver transplantation has excellent survival rates (>90% at 5 year) [39,40].

### Extra-hepatic manifestations

#### Kidney

The main differentiating feature between PCLD and ADPKD is the presence of polycystic kidneys. While this is the primary lesion in ADPKD, renal disease is absent in PCLD [27]. Disease progression depends on genotype, but also on environmental factors [25]. The majority of adult ADPKD patients develop enlarged kidneys and end-stage renal disease. In contrast, few renal cysts may be present in 28-35% of PCLD patients, but renal failure does not occur [15,19].

In contrast to PCLD, ADPKD is a multi-systemic disorder. Hypertension is one of the first signs of renal disease development and is related to progressive kidney enlargement and loss of renal function [41,42]. It is still unclear whether early anti-hypertensive treatment prevents renal function decline [43]. The most common feature is abdominal/flank pain due to pressure symptoms and stretching of the renal cyst wall. ADPKD patients are also at-risk for other renal complications such as hematuria, urinary tract infections and kidney stones [21,41].

#### Cardiovascular system

ADPKD patients may develop hypertension, intracranial aneurysms (ICA), arterial aneurysms and several cardiac valvular abnormalities [44]. Early assessment of cardiovascular risk factors in ADPKD is advised, especially in young ADPKD patients [45,46].

Mitral valve prolapse has a higher prevalence in ADPKD up to 25–41.2% compared to 0–10.5% in PCLD [19,27,47,48]. Other important connective tissue abnormalities such as aortic root dilatation and abdominal aorta aneurysm (AAA) have been reported in ADPKD [49]. If the family history is positive, screening of unaffected family members by MR-angiography is recommended [50,51].

#### Non-renal extra-hepatic cysts

The phenotype of PCLD is mainly restricted to the liver. Extensive radiological imaging of ADPKD patients have demonstrated that there are cysts in other abdominal organs such as the pancreas (9%) or seminal vesicles of the testis (43%) [52,53]. These cystic manifestations symptomatically silent.

Arachnoid cysts is central nervous system manifestation that is seen in 8% of ADPKD patients. This condition may occasionally lead to a subdural hematoma [50].

#### Abdomen

Abdominal wall hernias may be present in PLD patients. Clinical series have suggested that (para)umbilical and inguinal hernias may be seen in up to 15-45% of ADPKD patients which may be explained by chronic compression due to high liver and kidney volumes [27,41,54]. Early reports have suggested a higher prevalence of colonic diverticuli in PLD, but upon scrutiny there was no evident association or increased risk for diverticular disease [27,55].

## Etiology

### Germline mutation

Genetic analyses of both PCLD genes *PRKCSH* [OMIM*177060] and *SEC63* [OMIM*608648] may confirm the clinical diagnosis and differentiate it from other PLD (Table 1) [56-58]. These genes encode for the (glyco)proteins hepatocystin and Sec63p [59]. Both proteins are located within the endoplasmic reticulum (ER) and are responsible for quality control and translocation of glycoproteins into the ER [60]. Since about 16-22% of PCLD patients harbor a pathogenic variant, PCLD is assumed to be genetically heterogeneous and other loci should be nvolved. There is no clear genotype-phenotype association [15,61].

**Table 1:** Genetic and diagnostic determinants of PCLD and ADPKD

	**Polycystic liver disease(PCLD)**	**Autosomal dominant polycystic kidney disease (ADPKD)**	**Reference**
**Genotype**			
Cytogenetic gene location (mutation frequency%)	Chr.19p13.2: *PRKCSH* (15%)	Chr.16p13.3: *PKD1* (75.7%)	[62,63]
	Chr.6q21: *SEC63* (5.7%)	Chr.4q21: *PKD2* (13.4%)	
Mutation (type; N,%)	25 ***PRKCSH*** 22 ***SEC63***	980 ***PKD1*** 193 ***PKD2***	[HGMD]
*missense*	4 (16%) 6 (27.3%)	250 (25%) 29 (15%)	
*splice site*	4 (16%) 3 (13.6%)	77 (7.8%) 32 (16.6%)	
*insertion/deletion*	10 (40%) 7 (31.8%)	440 (45%) 80 (41.5%)	
*indel*	1 (4%) -	13 (1.4%) 7 (3.6%)	
*nonsense*	6 (24%) 6 (27.3%)	202 (20%) 45 (23.3%)	
*complex rearrangement*	-	8 (0.8%) -	
Gene product; protein localization	**Cholangiopathy**	**Ciliopathy**	[13,60]
	Hepatocystin/ glucosidase II-β subunit; ER	Polycystin-1 (TRPP1); primary cilium, tight junction, extracellular matrix, ER	
	Translocation protein SEC63 homolog;		
		Polycystin-2 (TRPP2); primary cilium, tight junction, extracellular matrix, ER	
	ER, membrane complex		
Protein function	Proper protein folding and protein quality control	PC-1 and PC-2 form a mechanosensor complex on the primary cilium	[13,60]
	Posttranslational protein transport	PC-1 for signaling detection, PC-2 is a TRP channel for calcium influx	
**Predominant phenotype**			
**Liver features**	Positive family history with:	Most common extra-renal manifestation: 83% with a polycystic liver (>20 hepatic cysts)	[14,64]
*PCLD diagnostic criteria:*			
*Clinical practice:*	<40 years and ≥1 hepatic cyst		
	≥40 years and ≥4 hepatic cysts		
	30-70 years and polycystic liver (>20 hepatic cysts)		
**Kidney features**	Incidental finding without renal disease: 28-35% with renal cystogenesis	Positive family history with unknown genotype:	[15,19,65,66]
*ADPKD diagnostic criteria:*			
		15-39 years and 3 renal cysts^#^	
		40-59 years and 2 renal cysts*	
		≥60 years and 4 renal cysts*	
		Negative family history:	
		<30 years and 5 renal cysts^$^	
		30-60 years and 5 renal cysts^$^	
		>60 years and 8 renal cysts^$^	

Description of the data: A detailed overview of genotype characteristics and diagnostic criteria for PCLD and ADPKD phenotype assessment.

^#^ unilateral or bilateral.

* in each kidney.

^$^ bilateral.

Mutations in the *PKD1* gene [OMIM*601313] or *PKD2* gene [OMIM*173910] are responsible for renal cyst initiation in >90% of the cases [62]. Several ADPKD families possess no mutations on *PKD1* or *PKD2*[67,68]. It is hypothesized that these cases are linked to another (yet unidentified) *PKD3* gene [OMIM*600666] [69]. *PKD1* gene carriers have a higher prevalence of hypertension, complications and a higher risk of progressive renal failure compared to *PKD2*. Renal failure occurs at a much earlier age in *PKD1* carriers compared to *PKD2* carriers [41].

### Second-hit hypothesis

PLD patients have a heterozygous germline mutation and it is hypothesized that cysts arise through functional loss of the second allele [59]. This is the rate-limiting step in the formation of cysts. Secondary, somatic hit mutations have been identified in *PKD1* or *PKD2* genes in liver and kidney tissues from ADPKD patients [70,71]. Similarly, loss-of-heterozygosity of the *PRKCSH* or *SEC63* allele is present in PCLD cyst tissues. This is fitting with the second-hit-model for tumorgenesis which dictates that the combination of a germline and somatic mutations result in inactivated protein in target tissues [72,73].

### Modifier genes and environmental factors

Mouse models suggest that additional genes may lead to hepatic and renal cystogenesis [6]. It has been shown that *HNF-1*β mutations affect disease progression and outcome in ADPKD [25,74].

Once a cyst have been formed, progression to clinical significant disease requires other mechanisms [8]. Cyst fluid contains serum proteins but also cytokines and growth factors which contribute to cyst formation [75,76]. Expression of estrogen hormone receptors in cyst epithelium may trigger growth advantages [77].

### Diagnosis

VMC is a radiological diagnosis [9,10]. Ultrasonography shows multiple hyperechoic areas in the subcapsular region. The comet-tail sign is a special form of reverberation artifact in detection of small cysts. This sign appears as a trail on the image if small calcific or highly reflective foci are interrogated on ultrasound [5]. MRI is preferred above CT-scanning as it readily shows multiple hyperintense focal lesions on T2-imaging [9].

**Figure 2:**
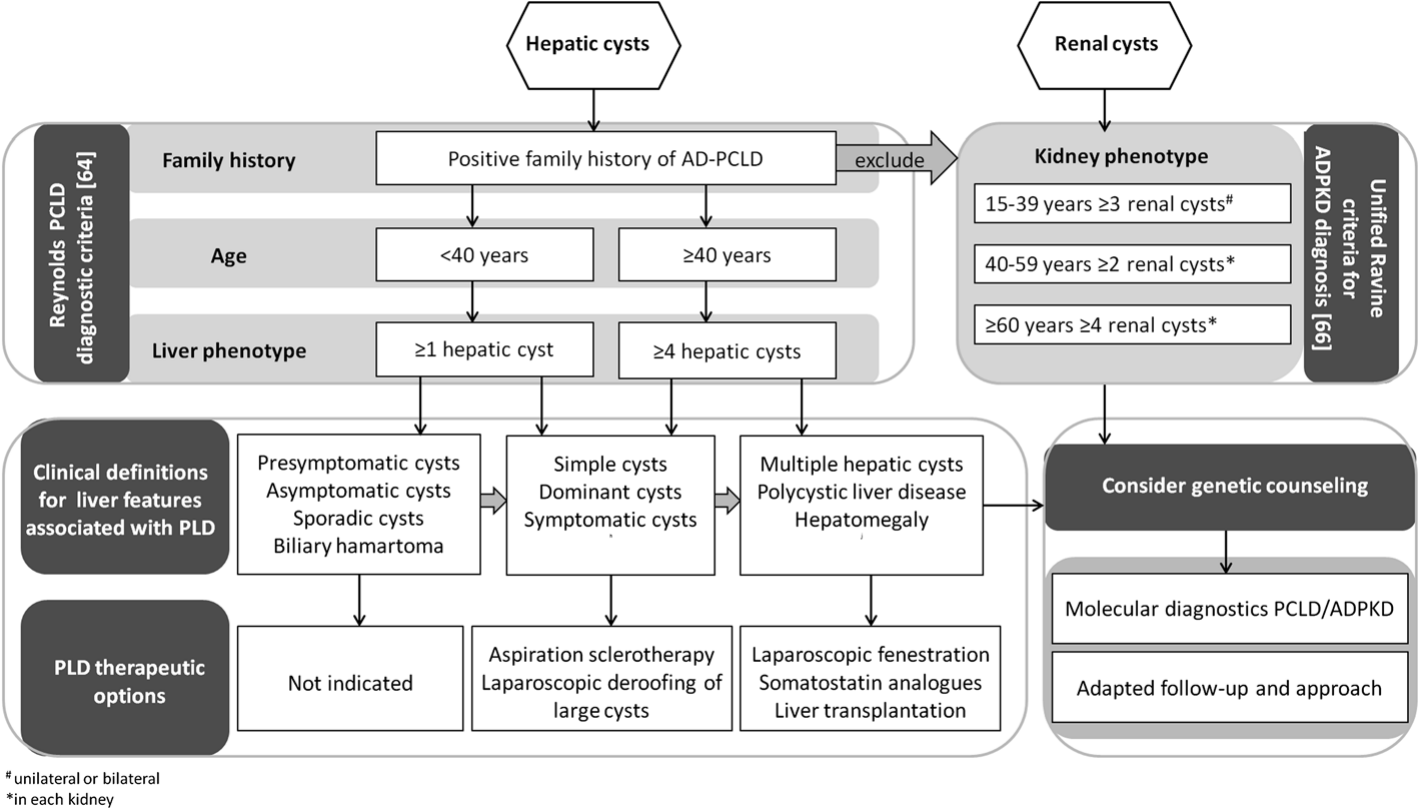
**Comprehensive algorithm for diagnosis, management and genetic counseling in PCLD and ADPKD.** The diagnostic criteria for PCLD and ADPKD compromises family history and age-related liver or kidney phenotype respectively [64,66]. PLD therapy is individually decided according to number, distribution and size of hepatic cysts [78]. Genetic counseling has an important role in symptomatic individuals with a positive family history for hepatic and/or renal cystogenesis in order to differentiate PLD and clinical management.

Current diagnostic criteria in PCLD and ADPKD rely on the age-related cystic liver phenotype with a positive family history of autosomal dominant inheritance (Figure 2) [64-66]. Ultrasonography of the liver and kidneys is usually the first modality used to assess a cystic phenotype. The unified Ravine criteria for diagnosis of ADPKD relies on counting the number of renal cysts in at-risk individuals [65,66].

A single laboratory test that is of discriminative value for PLD is lacking. The synthetic liver function is usually preserved in PLD. Mild elevations of γ-glutamyltransferase sometimes combined with elevated alkaline phosphatase values may be detected [15,19].

### Differential diagnosis

PLD is a non-communicating biliary tree disorder and should be differentiated from other neoplastic, infectious and traumatic conditions [4]. The diffusion and variable small-sized hyperechoic structures in VMC are important to differentiate from hepatic metastasis or microabcesses [9].

In general, differentiation from communicating biliary tree disorders is important for management and prognosis. Bile duct ectasias or cystic dilatations belong to connected intrahepatic cystic diseases. These features are detected in ARPKD, Caroli disease (CD) and Caroli syndrome (CS). PLD is characterized by intrahepatic disease, a more late onset of disease in adulthood and absence of congenital hepatic fibrosis (CHF).

### Autosomal recessive polycystic kidney disease

Young and adult ADPKD patients are difficult to distinguish with other hepatorenal fibrocystic diseases such as ARPKD and other ciliopathies [8]. ARPKD has an incidence of 1/20,000 live births (ORPHA731; prevalence of 1.2/100,000) and a high peri-natal lethality. The predominant phenotype includes perinatal renal cysts and CHF [12]. Presence of cystic dilatation of intrahepatic biliary tree may be confused with disconnected hepatic cysts [17]. The gene product polyductin is located at the primary cilium suggesting molecular similarities with the ciliopathy ADPKD [8].

### Caroli disease and Caroli syndrome

CD is characterized by saccular, cystic dilations of the more larger intrahepatic biliary system. In CS large and small intrahepatic bile duct ecstasies are accompanied with CHF. CS has been typically associated with renal disease as in ARPKD [7]. The incidence of CD is approximately 1/1,000,000 births, but CS is more frequent (ORPHA53035; ~250 cases). Both ARPKD and CS have an autosomal recessive inheritance pattern [12].

### Genetic counseling in PCLD and ADPKD

**Figure 3:**
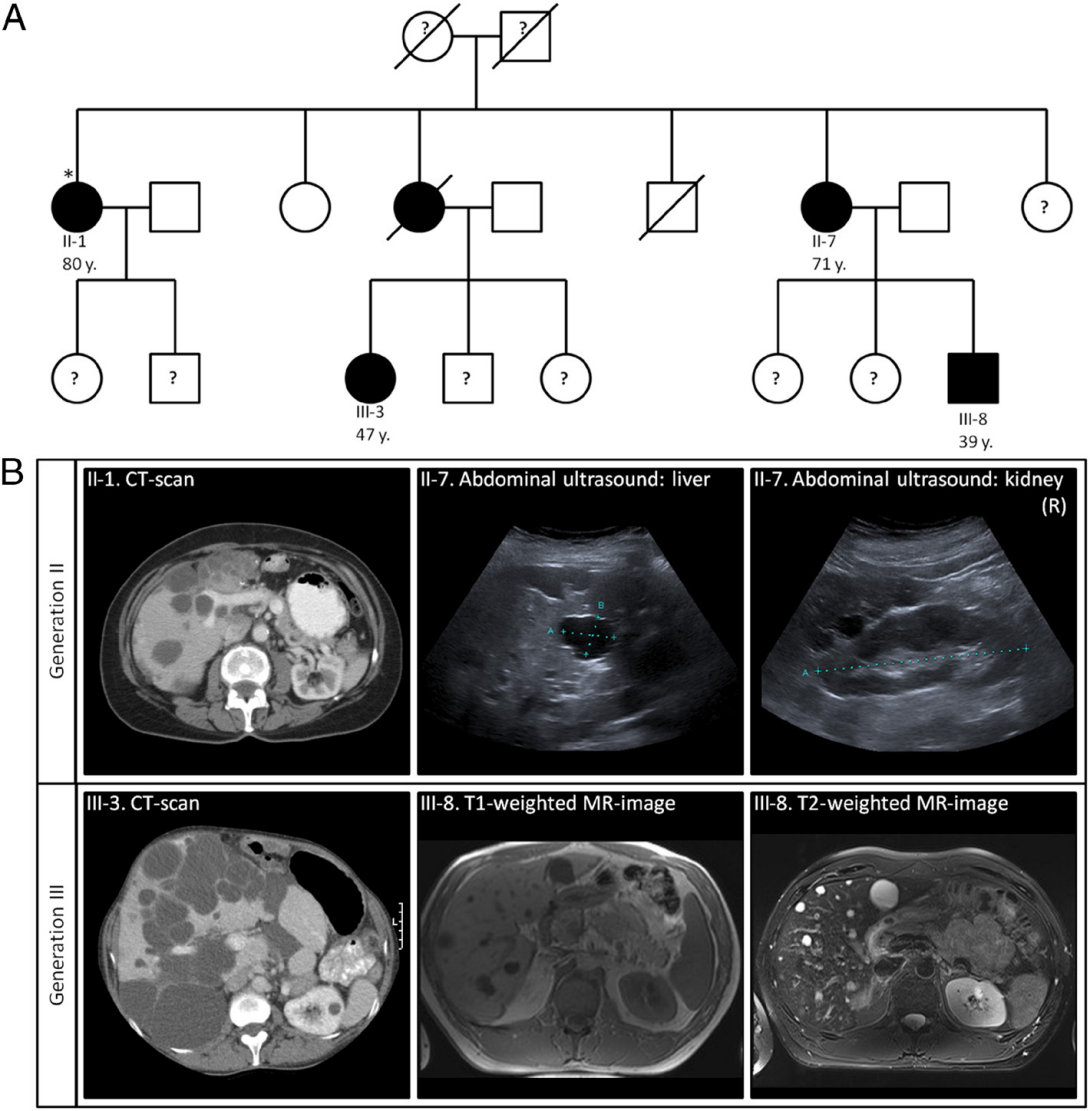
**Intra-familial clinical heterogeneity in PCLD.** Description of the data: **(A)** Pedigree of a PCLD family with *PRKCSH* gene mutation *c.374_375delAG* in affected individuals. The index patient (*) has 4 members with symptomatic PLD. Although the family history is positive, family members frequently are asymptomatic carriers or the liver phenotype remains unknown. **(B)** Axial CT-scanning, abdominal ultrasonography or MRI in 4 PCLD patients presented a variably number of hepatic cysts without renal disease.

Both PCLD and ADPKD have an autosomal dominant inheritance pattern and the recurrence risk is 50%. Genetic studies indicate an evident inter-familial clinical heterogeneity in PLD disease course among similar-aged patients. Secondly, intra-familial studies suggested a considerable phenotypic variability of hepatic cysts (Figure 3). Clinical asymptomatic or undiagnosed members contribute to underestimation of the actual disease prevalence [24,41,61]. It is estimated that the penetrance is ~80% [63].

These considerations raise the question whether it is appropriate to screen members or children at-risk. Counseling should include discussion about insurance, employment and psychological factors. Genetic counseling is recommended in severely affected PLD and may afford differentiation between ADPKD and PCLD [8,12].

Molecular diagnostics may assist the counseling process to establish a firm diagnosis in symptomatic patients and families. In particular ADPKD, determination of the responsible gene is useful for those who are at-risk in order to develop a strategy to prevent severe progressive disease events or complications [80]. If the family history for ICA is positive in an ADPKD family, screening of unaffected family members by MR-angiography is recommended [50,51,81]. Counseling or genetic testing is not advised in asymptomatic children [79].

### Management and prognosis

VMC is an asymptomatic condition without long-term consequences and treatment is not warranted. The primary aim of PLD therapy is to reduce symptoms by curtailing hepatic cyst development. The treatment of choice is driven by individual complaints [40]. Although the primary outcome measurement of PLD management is liver volume, assessment of symptoms associated with quality of life is an element for focus [82]. Therapeutic interventions are not warranted in asymptomatic patients.

The first advice in PCLD and ADPKD is to stop oral anticonceptives [20,78]. Although not formally investigated, the use other (non-systemic) contraceptives such as an intra-uterine device may be an acceptable alternative. Other guidelines are presented in Table 2[81,83]. Supportive management with analgesics is the first-line treatment in patients with acute or chronic abdominal pain and tenderness.

**Table 2:** Determinants and recommendations in severe PCLD and ADPKD

**Polycystic liver disease (PCLD)**		
**Organ**	**Determinant**	**Recommendations**
*Liver*	Female sex	Stop exogenous estrogen use in female patients [78]
	Aging [21,22]	Advise alternative contraceptive strategies
	Environmental factors associated with PLD disease course [21,22]:	
	-Prolonged oral/exogenous female steroid use: estrogens, contraceptive pill or (post-menopausal) hormonal replacement therapy	
	-Multiple pregnancies	
*Brain*	Similar recommendations seem appropriate for patients with isolated ADPLD, but more studies are required [19,79]	Indiscriminate screening is not recommended at present [44]
*Heart*	Similar as in the general population [47]	No recommendations
**Autosomal dominant polycystic kidney disease (ADPKD)**		
**Organ**	**Determinant**	**Recommendations**
*Kidney*	Environmental factors associated with renal cyst growth [83]:	Avoid (excessive) caffeine administration and nephrotoxic agents
	-caffeine	Smoking cessation
	-smoking	
	Influencing factors for renal cystogenesis [43,45]:	Hypertension [43,45,46,81]:
	-hypertension (≤35 years) - renal infection	-Routinely standardized blood pressure measurement
	-proteinuria - total kidney volume	
	-hematuria (<30 years) - male sex	-Elektrocardiogram in hypertensive patients for LVH assessment
	-urinary tract infection - low birth weight	
	-kidney stones - aging	-Plasma LDL cholesterol control; urinary albumin excretion; left ventricular mass index calculation
		-Angiotensin converting enzyme inhibitors and/or angiotensin receptor blockers
		Dietary protein and salt restriction
		Sufficient daily fluid intake
	*PKD1* gene mutation have a more severe disease course and earlier onset of end-stage renal disease compared to *PKD2* carriers [41]	Molecular diagnostics [24]
*Liver*	Female sex	Stop exogenous estrogen use in female patients [78]
	Aging [21,22]	Advise alternative contraceptive strategies
	Environmental factors associated with PLD disease course [24,25]:	
	-prolonged oral/exogenous female steroid use: estrogens, contraceptive pill or (post-menopausal) hormonal replacement therapy	
	-multiple pregnancies	
	Renal function/glomular filtration rate [23]; in particular females [43]	
*Brain*	Patients at risk:	Patients with reasonable estimated life expectancy: periodic 3–5 years MR/CT-angiography screening [51]
	-positive family history of (ruptured) ICA or stroke <50 years old	
	-previously ruptured ICA	Surveillance/rescreening after negative results in patients with a positive family history: 5–10 years (high-to low-risk) [13,81]
	-warning symptoms: unusual headaches	
	-high-risk occupation (for example: airline pilot)	
	preparation for major elective surgery (for example: kidney transplantation) [13,51]	
		Smoking cessation
		Blood pressure control
	The position of the mutation in *PKD1* is predictive for development of intracranial aneurysms [80]	
		Hyperlipidemia control [51]
		Molecular diagnostics [80]
*Heart*	Screening is indicated [13,45]:	Echocardiography [13,45]
	-a murmur or systolic clicks are detected on examination	
	-positive family history of thoracic aorta dissection	
*Aorta*	ADPKD patients receiving hemodialysis [49]	AAA: routine screening of the aortic size, using CT or abdominal ultrasonography [49]
	Similar as in general population for AAA [81]:	1-time screening with abdominal ultrasonography [81]
	-Male between the ages of 65–75 and smoked >100 cigarettes in a lifetime	
	-Male >60 years and a family history of AAA	

This scheme indicates distinct factors that are related to physical health and disease progression in PCLD and ADPKD patients. These clinical elements listed per organ may be valuable for consideration in management. Patients’ mental condition and health-related quality of life should be assessed by standardized evaluation of symptoms in all PLD patients [82].

**Figure 4:**
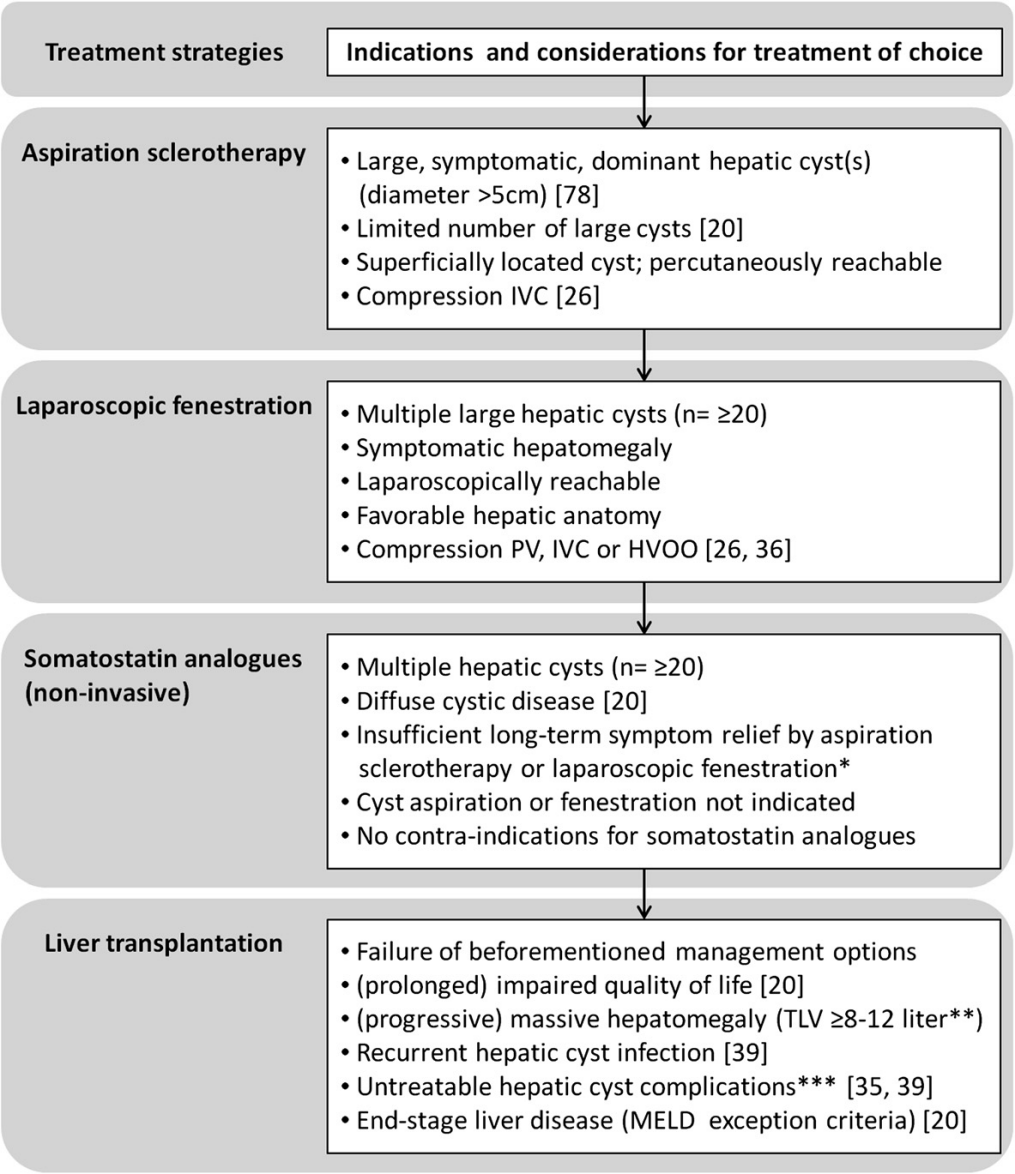
**Indications and considerations for treatment strategies in symptomatic PLD.** Schematic overview of relevant indications and considerations in PLD management. Patient characteristics and liver phenotype such as severity of clinical symptoms, age, (surgical) history, degree of hepatomegaly, and number, size and location of hepatic cysts are essential parameters for this decision. In general, the choice of procedure is an individual process in consultation with the patient. Severely affected individuals are patients with massive hepatomegaly, refractory symptoms, PLD-related complications and/or end-stage liver failure. Care and follow-up of these patients should be managed with caution. *Notification: Results about treatment efficacy of prolonged or long-term somatostatin analogue use is unknown. **Arbitrary total liver volume (TLV), because this outcome measurement is related to other parameters such as height, total body surface and sex. ***For example: recurrent hepatic cyst infection, HVOO.

The different invasive approaches with possible beneficial outcomes in independent studies include aspiration sclerotherapy, laparoscopic cyst deroofing or liver transplantation [39,40,84]. Current indications and considerations for invasive treatment are presented in Figure 4.

Treatment of portal hypertension and ascites are not different from that in patients with other causes. In a selected patient group were invasive procedures such as a vascular or bile duct stent placement optional for decompression of the portal vein, inferior vena cava or bile duct, or in HVOO treatment [37,85]. In general, management of vascular and bile duct complications due to hepatic cystogenesis consists of relieving the obstruction in order to improve venous and biliary drainage [26,35]. Although no concise guidelines are available, stents may give temporal symptom relief of portal hypertension, ascites and jaundice. A porto-systemic shunt may be indicated in presence of acute thrombosis or vascular compression to establish patent hepatic and portal venous flow, but should be weighed against possible complications. The primary aim is to ameliorate symptoms by cyst decompression in these advanced cases. This is often achieved by final treatment strategies including liver resection, hepatic fenestration procedures or liver transplantation [33].

Recent development of pharmacological options opened up new treatment strategies for severe PLD patients. Long-term follow-up studies with somatostatin analogues demonstrated that these agents consistently lower total liver volume in PLD patients [86,87]. A recent meta-analysis reported that somatostatin analogues is particularly effective in young females [88].

## Conclusion

PLD compromises a clinically heterogeneous liver phenotype identified in VMC, ADPKD and PCLD patients. Massively enlarged livers are present in a subset of ADPKD and PCLD. Genetics and environmental factors such as exogenous estrogen intake and number of pregnancies contribute to disease progression. A considerable intra-familial variability in liver phenotype and extra-hepatic features makes screening modalities uncertain in PCLD. Evaluation of PLD-related symptoms and quality of life are necessary to decide beneficial management.

### URL

Human Gene Mutation Database for Human Genetics Research; HGMD® Professional 2013.3 - 27^th^ September 2013; http://www.biobase-international.com/product/hgmd.

## Abbreviations

AAA: Abdominal aorta aneurysm; ADPKD: Autosomal dominant polycystic kidney disease; ARPKD: Autosomal recessive polycystic kidney disease; CD: Caroli disease; CS: Caroli syndrome; CT: Computer tomography; CHF: congenital hepatic fibrosis; DPM: Ductal plate malformation; ER: Endoplasmic reticulum; HVOO: Hepatic venous outflow obstruction; ICA: Intracranial aneurysm; IVC: Inferior vena cava; LVH: Left ventricle hypertrophy; MELD: Model for end-stage liver disease; MRI: Magnetic resonance imaging; PC1: PC2 polycystin-1, -2; PCLD: Isolated polycystic liver disease (autosomal dominant); PKD1: PKD2 polycystic kidney disease-1, -2; PLD: Polycystic liver disease; PRKCSH: Protein kinase C substrate 80 K-H (80-kDa protein, heavy chain); SEC63: Saccharomyces cerevisiae homolog 63; VMC: Von Meyenburg complexes.

## Competing interests

Authors declare that they have no competing interests.

## Authors’ contributions

WC researched the data and drafted the manuscript. WC and JD contributed to the content and wrote the article. JD is responsible for critical revision of the content. Both authors read and approved the final manuscript.
